# Harnessing Citizen Science to Support Nature-Based Flood Management: A Case Study of Photo Post Engagement in the Limb Brook Catchment, Sheffield, UK

**DOI:** 10.1007/s00267-026-02464-7

**Published:** 2026-05-22

**Authors:** Melissa M. Lacey, Cedric Andreasen, Jackie Lowe, John McGlinchey, Patrick Sheard, Claire Bithell, Samuel Lilleker, Martin Reed, Roger Nowell, Rachel Schwartz-Narbonne, Rebecca Sharpe

**Affiliations:** 1 School of Biosciences and Chemistry, Sheffield Hallam University, Sheffield, UK; 2 South Yorkshire Partnerships and Strategic Overview Team, Flood and Coastal Erosion Risk Management of the Environment Agency, Sheffield, UK; 3 Sheffield and Rotherham Wildlife Trust, Sheffield, UK; 4 Roger Nowell at Sheffield City Council, Sheffield, UK; 5 Institute of Law and Social Sciences, Sheffield Hallam University, Sheffield, S1 1WB UK

**Keywords:** citizen science, nature-based solutions, photo post, community engagement

## Abstract

Nature-based solutions (NBS) are increasingly promoted in climate adaptation strategies to mitigate flood risks and enhance ecosystem resilience. However, the monitoring and evaluation of such interventions remain a challenge due to resource limitations. This paper evaluates a citizen science-based monitoring approach in the Limb Brook catchment (Sheffield, UK), where public photo submissions were used to assess the effectiveness of NBS interventions such as leaky dams and attenuation ponds. Over a 26-month period, over 4500 photographs were collected from 15 fixed photo points. The analysis demonstrates high initial engagement, varied spatial interaction across sites, and the potential for real-time detection of intervention failures. We critically examine how public participation in environmental monitoring can support adaptive flood risk management while highlighting barriers related to data infrastructure, privacy regulations, and long-term community involvement. This study underscores the value of integrating citizen science into national and local policy frameworks for environmental governance.

## Introduction

Flooding represents one of the most pressing and costly environmental challenges facing urban and peri-urban areas globally. Driven by land-use change, population growth, and the intensifying impacts of the climate crisis, flood frequency and severity are increasing in many regions. There is growing evidence that flood frequency and impact are increasing globally, driven by a combination of climate change and land-use dynamics (e.g. Guha-Sapie et al. 2015; IPCC 2023). The Intergovernmental Panel on Climate Change (IPCC) Sixth Assessment Report highlights flooding as one of the most acute climate-related risks facing human settlements, with urban areas particularly exposed due to the prevalence of impermeable surfaces, channel modifications, and concentrated populations (IPCC 2023). Traditional ‘grey’ flood defences such as embankments, levees, and concrete channels, while effective in the short term and in their specific location, are expensive to maintain, carbon-intensive to construct, and often maladaptive under conditions of climatic variability. Moreover, they tend to transfer rather than reduce risk, displacing flood impacts downstream and failing to address the root causes of hydrological instability (Pamungkas and Purwitaningsih 2019). In response, recent decades have seen a growing recognition of the need for more adaptive, sustainable, and integrated approaches to flood risk management that work with natural processes rather than against them. This paradigm shift has catalysed the emergence of Nature-Based Solutions (NBS) as a central concept in both environmental policy and practice.

### Nature-Based Solutions: Concepts and Policy Drivers

Nature-based Solutions (NBS) address societal challenges through actions to protect, sustainably manage, and restore natural and modified ecosystems, benefiting people and nature at the same time (International Union for Conservation of Nature 2020).

Within the domain of flood risk management, NBS are frequently implemented through Natural Flood Management (NFM) measures. Within the UK setting, NFM focused on seeking to slow, store, and filter water within catchments. Typical interventions include wetland restoration, riparian woodland planting, peatland rewetting, floodplain reconnection, attenuation ponds, and engineered leaky dams. These measures can attenuate flood peaks downstream while improving water quality, sequestering carbon, enhancing habitats, and providing recreational and aesthetic value (Rameshwaran et al. 2024).

At the policy level, NBS have gained significant traction across international and national frameworks. The European Union’s Green Deal promotes NBS as tools for climate adaptation and biodiversity enhancement, while the United Nations Sustainable Development Goals (SDGs), notably SDG 13 (Climate Action) and SDG 15 (Life on Land), explicitly advocate for ecosystem-based approaches to environmental management (European Commission 2019). The International Union for Conservation of Nature (IUCN) has formalised these principles through its Global Standard for NBS, providing a framework for design, implementation, and evaluation (International Union for Conservation of Nature 2020).

In the United Kingdom, the *National Flood and Coastal Erosion Risk Management Strategy for England* (Environment Agency 2020) encourages NBS as complementary to conventional infrastructure, emphasising catchment-scale planning, integrated management, and community participation (Newson and Lewin 2025). However, despite this growing institutional support, widespread implementation and evaluation of NBS remain constrained by practical and epistemic challenges, particularly concerning the robust monitoring of their effectiveness (European Environment Agency 2021).

### Monitoring NBS Effectiveness: Challenges and Gaps

The appeal of NBS lies in their ability to deliver multiple co-benefits—reducing flood risk while enhancing ecological integrity and social well-being. Yet despite their conceptual attractiveness, empirical evidence regarding their hydrological effectiveness and long-term performance remains limited. Case studies indicate that measures such as leaky barriers, floodplain reconnection, and attenuation ponds can reduce peak flows during moderate rainfall events, but their performance under extreme or compound flooding remains less certain (Birkinshaw and Krivtsov 2022; Newson and Lewin 2025; van Leeuwen 2024; Villamizar et al. 2024). Similarly, many ecological and carbon sequestration benefits accrue gradually, requiring long-term observation to capture fully (Seddon et al. 2021).

The challenge, therefore, lies not only in implementing NBS but in evaluating their performance under diverse hydrological, ecological, and social conditions. In the UK, funding models typically support the design and installation of NBS but rarely include dedicated budgets for medium- or long-term monitoring, maintenance, or evaluation. For example, the UK Flood and Coastal Erosion Risk Management (FCERM) scheme commits to £4.2 billion investment between 2026 and 2029 to “reduce risk from, and increase resilience to, flooding and coastal erosion”. FCERM policy provides funding guidelines for Standalone NFM project interventions; however, it explicitly states, “to receive funding a standalone NFM project should also have maintenance plans with funding options in place, as this will not be funded through the FCERM investment programme” (UK Government 2026). Funding for maintenance and monitoring of NFM intervention then falls to the landowner, unless an organisation adopts the intervention as an asset to manage, or is encompassed in private investment models such as Riverlution (River Stewardship Company 2026). This institutional limitation hinders the accumulation of robust, longitudinal data necessary to build scientific confidence and policy legitimacy.

Monitoring is fundamental for both scientific credibility and societal acceptance of NBS. Without robust monitoring, the expansion of NBS risks being undermined by scepticism regarding their effectiveness compared to engineered defences due to a lack of evidence of their impact (European Environment Agency 2021; UNEP 2026). Conventional hydrological monitoring methods such as flow gauges, rainfall loggers, and detailed modelling remain standard for quantifying impacts. However, these methods are resource-intensive, require specialist expertise, and are often spatially restricted to a limited number of locations (Castellari et al. 2021; Newson and Raven 2025). Recent advances have begun to address some limitations in environmental monitoring through a range of emerging approaches. These include low-cost sensing technologies, which enable distributed and continuous data collection (Gobatti et al. 2022); UAV-based monitoring (Hill 2025), which supports high-resolution spatial analysis of hydrological and geomorphological processes. While these approaches have expanded monitoring capabilities, they often remain limited in capturing localised, experiential, and long-term observations, struggling to reconcile high-tech spatial data with qualitative, community-scale perspectives. In small tributary catchments, which are numerous across river basins, establishing and maintaining comprehensive professional monitoring networks is rarely feasible (Jones et al. 2025). Furthermore, formal monitoring frameworks are typically oriented toward regulatory compliance rather than adaptive management, leaving substantial gaps in understanding how NBS perform in real time or across varying climatic and ecological conditions (Horneman et al. 2026).

These limitations have created a persistent practice and knowledge gap in the evaluation of NBS. While demonstration projects can evidence effectiveness at a site scale in the short term, there remains a broader deficit of long-term, spatially distributed monitoring data (Sowińska-Świerkosz and García 2021; Newson and Raven 2025; Rödl and Arlati 2022). This limitation constrains not only scientific understanding but also public and political support for NBS, which depend on credible, visible evidence of benefits (Nesshöver et al. 2017). Addressing this gap requires innovative, scalable, and cost-effective monitoring approaches that can complement professional networks and support the generation of longitudinal datasets. Emerging initiatives such as the Catchment Systems Thinking Cooperative (CASTCo) in the UK exemplify efforts to develop standardised, community-based monitoring frameworks to address these challenges (CASTCo 2026).

### Citizen Science as a Tool for Environmental Monitoring

Citizen science encompasses the participation of non-scientific individuals and communities in scientific research; its roots can be found in the mid-1990s and in Irwin’s work *Citizen Science: A Study of People, Expertise, and Sustainable Development* (Irwin 1995). Citizen science projects now span scientific fields, from ecology (Green et al. 2023), and astronomy (Kyba et al. 2023) to public health (Rosas et al. 2022) and urban planning (Cappa et al 2022) with an estimated global value of over $2.5 billion annually for biodiversity citizen science alone (Theobald et al 2015).

Citizen science is attractive to scientists as it allows the generation of large datasets over broad spatial and temporal scales as well as the identification of specific traits (e.g. species) in large data sets (Johnston et al. 2020). There is growing evidence that involvement in some citizen science projects can increase the public’s understanding of specific topics (Bonney et al. 2015, Brossard et al. 2005, Crall et al. 2012).

Shirk et al. (2012) building on the work of Bonney et al. (1996), postulates three forms of citizen science: 1) Contributary: the public collects and contribute data and/or samples as requested by scientists, 2) Collaboratory: the public collects and analyses data to address research goals which are shared with the scientists and 3) Co-creative: where the public develops a study and work with input from scientists to address a question of interest or an issue of concern.

Contributory citizen science projects are the most common (Theobald et al. 2015), with many projects involving images, with the public taking photographs and these being analysed by scientists (Flowers et al. 2023, Harley and Kinesla 2022, Osawa et al. 2017) or images provided by scientists being analysed by the public (Green et al. 2023, Swanson et al. 2016).

In hydrology and water management, citizen science is less established but increasingly recognised (Butaert et al. 2014, Nardi et al. 2021, Newson and Raven 2025). Initiatives such as Riverfly monitoring in the UK (Moolna et al. 2020) and participatory mapping of flooding (Mazzoleni 2017, Mazzoleni et al. 2018) demonstrate the potential of public participation to provide early warnings, fill monitoring gaps, and raise awareness of water-related hazards. Emerging programmes such as CASTCo further aim to integrate citizen science into catchment-scale monitoring and decision-making frameworks (CaSTCO 2026). Research further suggests that citizen science can enhance environmental stewardship, as participation is associated with increased environmental knowledge, place attachment, and pro-environmental attitudes and behaviours (Fraisl et al. 2022; Phillips et al. 2019; Somerwill and Wehn 2022). For NBS, where local acceptance and stewardship are often prerequisites for long-term sustainability, citizen science offers both practical and social benefits (Jadeja et al. 2018, Landstrom et al. 2011, Landstrom et al. 2019).

### Research Gap: Citizen Science and NBS Monitoring

While citizen science has become a well-established component of biodiversity monitoring, its application to hydrological monitoring, particularly in relation to NBS, remains emerging and undeveloped (Newson and Raven 2025). Empirical studies examining how communities engage with NBS projects through citizen science approaches are limited, especially in the UK context. This gap is notable given the increasing policy emphasis on community participation and the practical need for cost-effective, scalable monitoring frameworks.

Recent reviews highlight persistent gaps in the systematic evaluation of citizen science applications in hydrological monitoring, particularly in relation to data quality frameworks and their operationalisation (Fraisl et al. 2022; Saleem et al. 2024). While the potential of citizen science to capture dynamic hydrological processes such as fluctuations in water levels, the performance of leaky barriers, and geomorphological change is increasingly recognised, there remains limited empirical evidence assessing its effectiveness in these contexts. Furthermore, relatively little attention has been given to how citizen-generated data might complement professional monitoring networks or be incorporated into adaptive management strategies (Newson and Raven 2025; Veness et al. 2025). Uncertainties also persist regarding the long-term sustainability of participant engagement (Koedel et al. 2024; Somerwill and Wehn 2022) and the reliability and operational suitability of crowd-sourced environmental data for management applications (Fraisl et al. 2022; Kelly-Quinn et al. 2023).

### Limb Brook Case Study: Policy Context and Project Setting

**Fig. 1:**
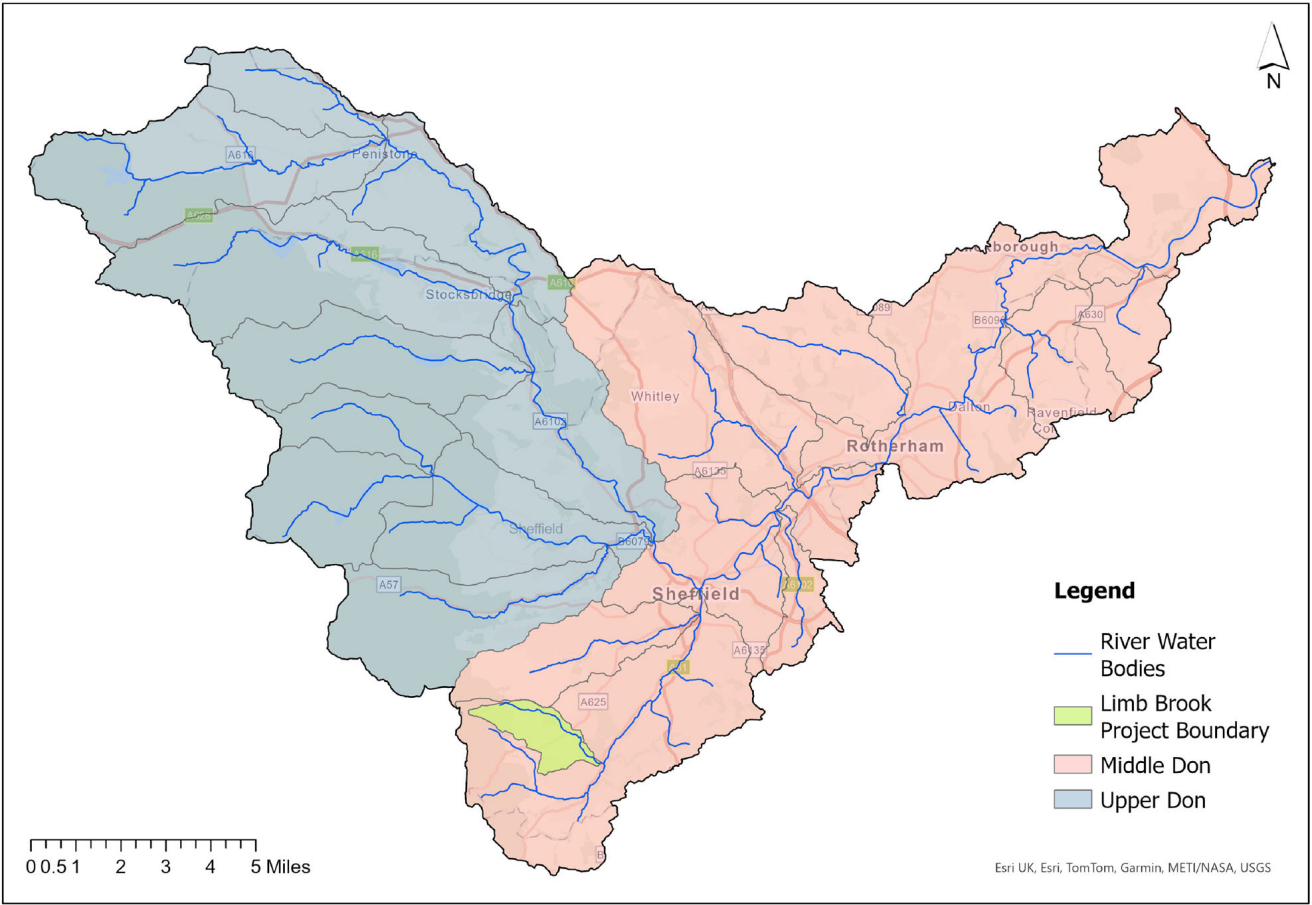
Don catchment map. Blue: Upper Don catchment, Red: Middle Don catchment, Green: Limb Brook Project Boundary

Sheffield (UK) has a long history of main river flooding, with records extending back as far as 1767. In June 2007 extensive flooding occurred, resulting in the loss of two lives and in excess of £570 million of flood damage (6.2% of Sheffield’s Gross Value Added). The Limb Brook sub-catchment (Limb Brook Valley, Sheffield (UK), centred around coordinates 53.35°N, 1.54°W) forms part of the River Sheaf, a tributary of the River Don (Fig. 1). Extending from upland moorland and plantation forestry to peri-urban green spaces and ancient woodland, the catchment typifies the mixed land uses common to many UK sub-catchments. Within the Limb Brook Valley project boundary, land use comprises 58% improved grassland, 16% coniferous woodland, 13% broadleaf woodland, 12% suburban and 1% mixed other. Historically, the Limb Brook has exhibited rapid surface runoff and flash hydrological responses during rainfall events, contributing to downstream flood risk in Sheffield.

These characteristics made the Limb Brook an appropriate demonstrator site within the wider *Upper Don Source to Sea* programme, which aims to integrate NBS across the Don catchment to mitigate flood risk and deliver wider ecosystem benefits.

**Fig. 2:**
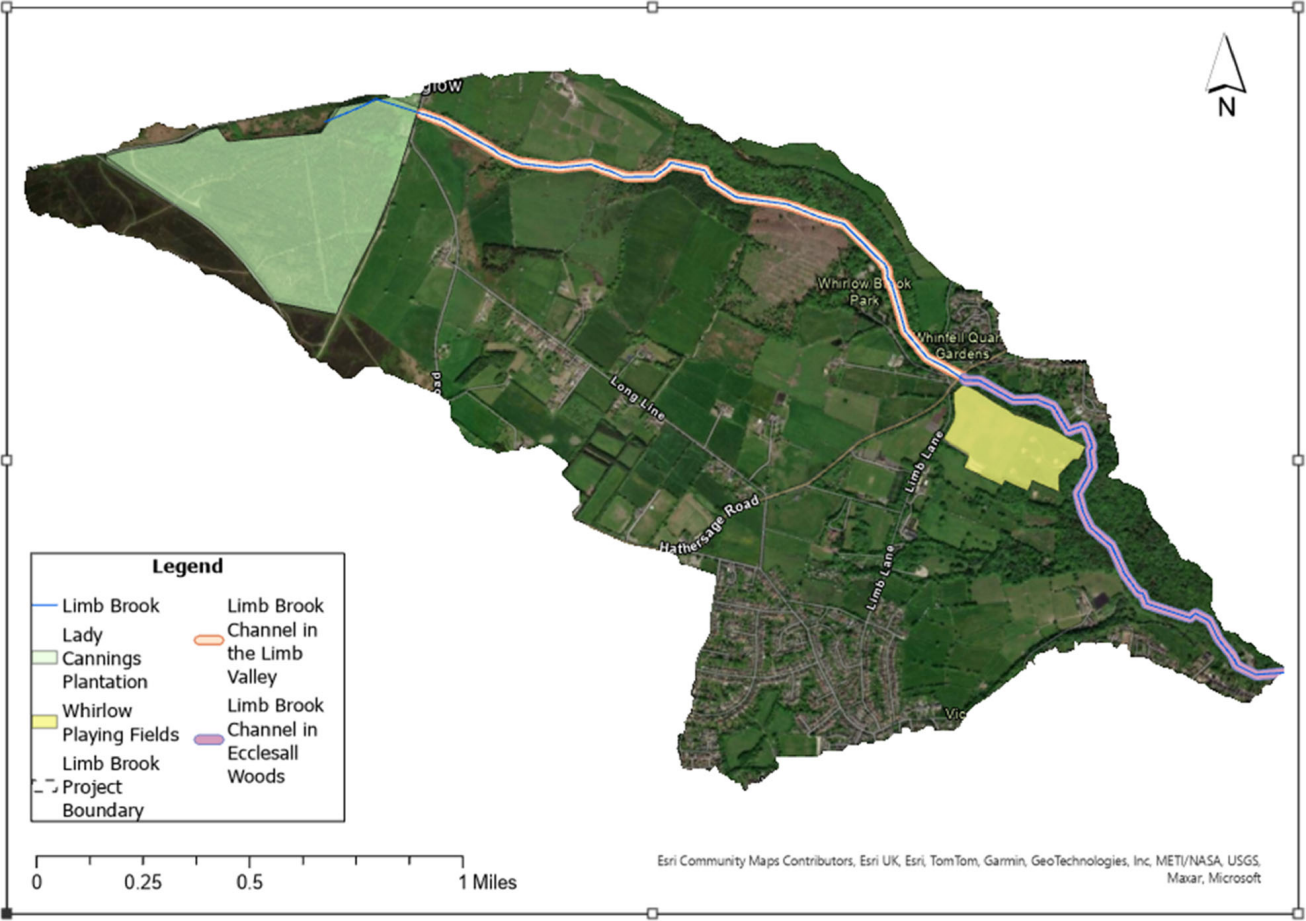
Limb Brook sub catchment. Green: Lady Cannings Plantation, Yellow: Whirlow Playing Fields, Orange: Limb Brook channel in the Limb Valley, Purple: Limb Brook channel in Ecclesall Woods

The project encompasses a series of Sheffield City Council (SCC)–owned parks, woodlands, and green spaces (Fig. 2). It was co-designed and delivered through a partnership between the Environment Agency (EA), SCC, and Sheffield and Rotherham Wildlife Trust (SRWT). Funded through the UK government’s accelerated capital works programme (2021–2022), as part of the Upper Don Source to Sea programme (Connected by Water 2026), the initiative reflects national priorities to invest in climate resilience and stimulate local economies in the post-pandemic recovery period. The project had three primary objectives: (i) to reduce surface runoff and slow river flows through NBS interventions; (ii) to engage local communities in flood risk awareness and stewardship; and (iii) to explore cost-effective alternatives to formal hydrological surveys for assessing intervention performance.

The project area was divided into four main zones (Fig. 2): Lady Cannings Plantation, the Limb Brook channel within the Limb Valley, Whirlow Playing Fields, and Ecclesall Woods. Each represents distinct land-use and hydrological characteristics, providing a valuable basis for comparative assessment of NBS performance.

**Fig. 3:**
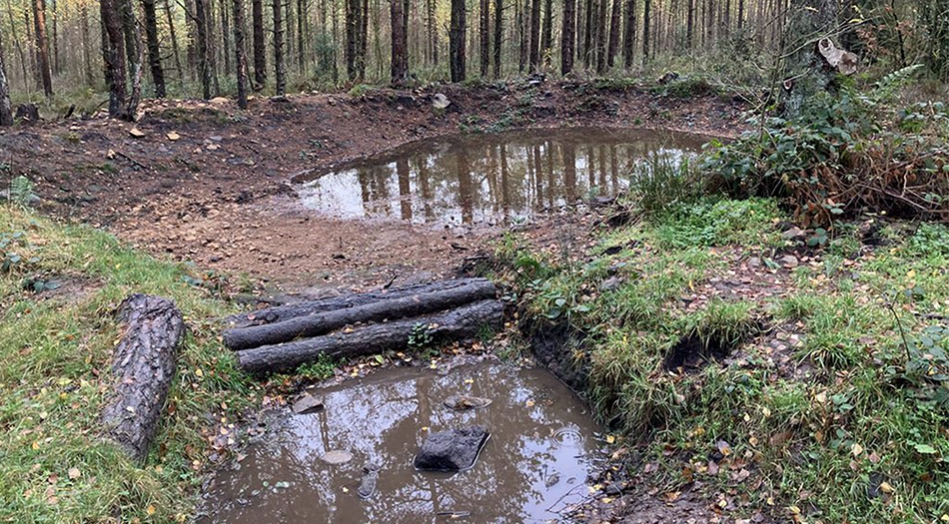
Lady Cannings Plantation attenuation pond with sediment trap

**Fig. 4:**
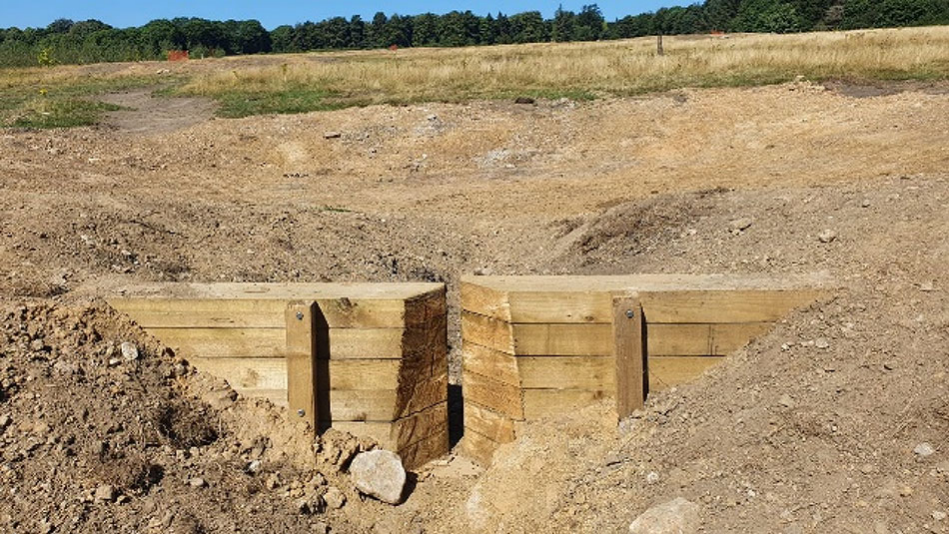
Whirlow Brook attenuation pond

At Lady Cannings Plantation, a 49-hectare coniferous woodland at the top of the catchment, approximately twelve attenuation ponds and associated sediment traps were installed to store runoff and reduce erosion along heavily used paths (Fig. 3). Within the Limb Valley and Ecclesall Woods, more than fifty leaky dams were constructed by volunteers and community groups such as the Friends of Whirlow to slow water movement through wooded channels and enhance temporary storage. At Whirlow Playing Fields, drainage systems were modified to restore surface hydrology through six attenuation ponds connected by swales (Fig. 4).

The leaky dam, attenuation pond and swale interventions combined are estimated to provide 2000 m^3^ storage, although a feature diverting flows into an existing basin at Lady Cannings Plantation is not included in this figure. Individual interventions were designed to trial different NFM approaches. For example, the three pairs of Whirlow Fields attenuation ponds were designed with different outflow controls and return periods: 1 in 1 year, 1 in 30 years, 1 in 100 years, respectively. The interventions are expected to last ten years, although an element of the project was to monitor how they perform over time and assess future maintenance requirements.

**Fig. 5:**
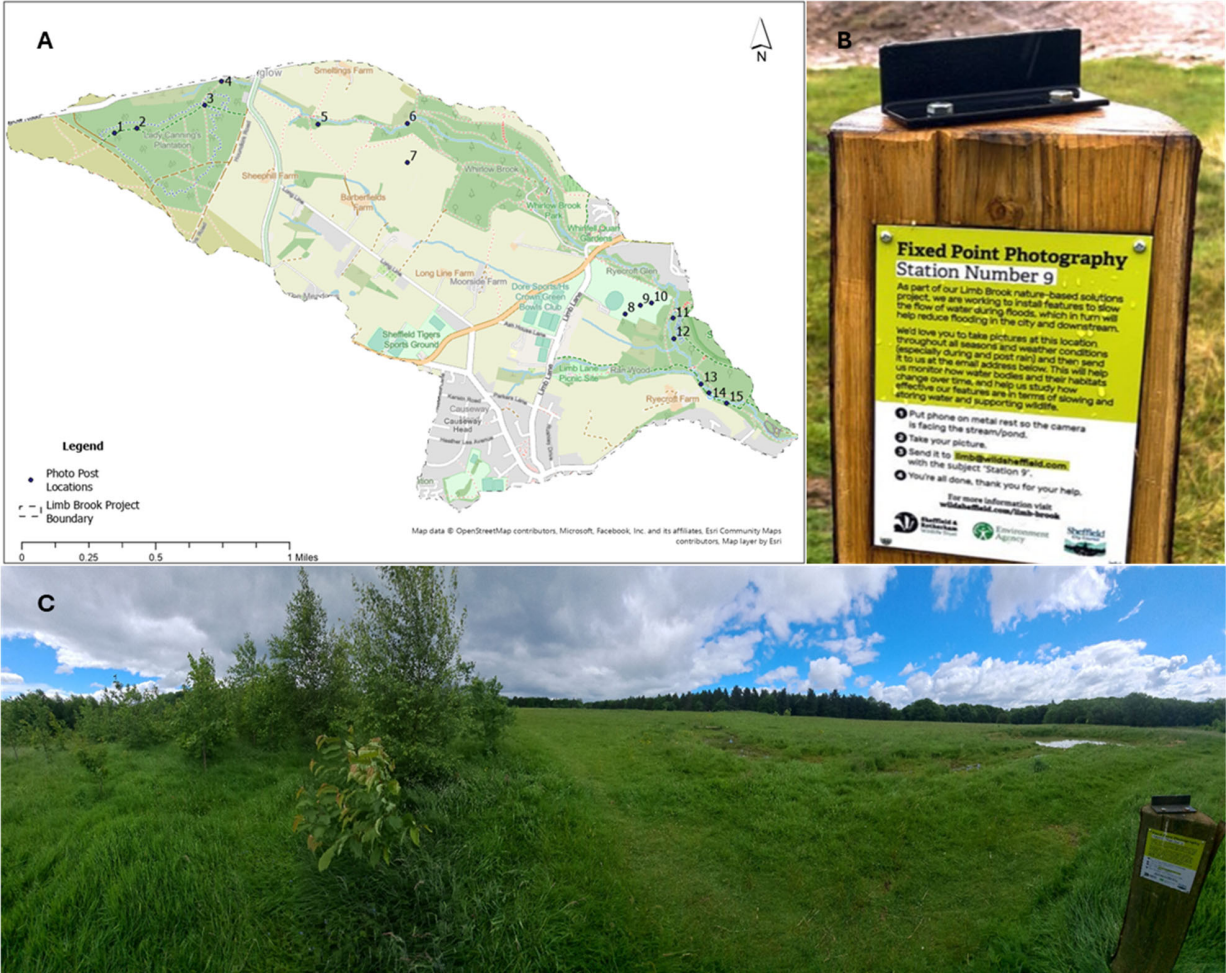
Photo posts in the Limb Brook catchment. **A** Location of photo posts within the Limb Brook Catchment, **B** photograph of post showing fixed point photograph point and **C** photo post in situ

A defining feature of the Limb Brook Demonstrator Project was the integration of citizen science as a participatory monitoring tool. Fifteen fixed photo posts were installed across the four project zones (Fig. 5), encouraging members of the public to take repeat photographs and submit them electronically to SRWT. The posts were positioned to capture key NBS features such as attenuation ponds, leaky dams, and stream channels both upstream and downstream of interventions. This approach sought to generate visual time-series data to complement professional maintenance monitoring of the NFM interventions undertaken by SRWT, while simultaneously fostering local engagement and stewardship.

By combining engineered natural interventions with citizen-led observation, the Limb Brook project exemplifies a hybrid approach to NBS implementation, integrating ecological design with participatory monitoring. It provides a testbed for examining both the scientific utility of citizen-generated imagery and the social dynamics of sustained community involvement in environmental management. The findings from this case study contribute to emerging debates on how participatory science can support the evaluation and long-term resilience of NBS in complex catchment systems.

### Research Aims

This paper addresses two central questions:

1. How effective are citizen science photo posts in monitoring the impacts of NBS interventions on hydrological processes, geomorphology, and biodiversity?
2. To what extent can such mechanisms sustain public engagement and contribute to participatory environmental governance?

## Methods

### Citizen Science Mechanism **Photo Posts**

Fifteen fixed-point photo posts were installed at strategic locations to capture seasonal and hydrological variation in NBS performance. Posts included signage inviting the public to take photographs and email them to a project inbox, with the title of the email corresponding to the number of the photo post. Photos collected in the email inbox were processed using a Google AppsScript (Lacey and Andreasen 2025), which saved attached photos and sorted them into folders based on the email subject line. The script required emails to contain the subject line ‘Station’ followed by a number 1–15. The script could only process emails with a single attachment that contained the correct subject line. Emails that did not conform to the sorting criteria were added to a separate folder for manual sorting. Each photograph’s metadata (date/time) was used to name photographs within a folder for each photo post using a Google AppsScript (Lacey and Andreasen 2025). Photographs without usable metadata were excluded from the study. Sorted photographs were visually screened within the folder to ensure the photos matched those of their designated site; indiscernible photos were removed. Initially, photographs were processed by SRWT within the Limb Brook Demonstrator Project’s initial funding. Subsequent photographs were processed by Sheffield Hallam University as part of the Natural Environment Research Council, BLUECEES (Growing Shoots Award) project.

### Data Processing and Analysis

Photographs submitted between May 2022 and June 2024 were automatically labelled and filtered using custom Google Apps Scripts. The number of unique users engaging with the posts was determined by anonymising and counting email addresses using an AppsScript (Lacey and Andreasen 2025). Unique users were sorted into categories for engagement based on the total number of emails sent during the analysis window (Short and sweet, 1–3 photos; Dabbler, 4–9 photos; Hardcore ≥10 photos). The number of labelled and sorted photos was extracted using an AppsScript (Lacey and Andreasen 2025). This was used to calculate the number of visits per month for each post. Monthly mean-rainfall data for the North of England was obtained from the Met Office to provide statistics for visits compared to rainfall. A local polynomial regression fitting was created from this data to account for the nature of the monthly aggregated data. This created a smoother relationship between rainfall and post visits.

Photo sequences were visually assessed to identify geomorphological changes, flooding, dam failures, and vegetation dynamics. The following impacts were identified: slowing of flow via attenuation ponds and biodiversity effects (Case Study 1); flow slowing by leaky dams and dam failure (Case Study 2); and geomorphological impacts (Case Study 3). Story timelines were created, for example, posts that exhibited significant geological changes during the study. Keyframes were added at dates where photos showed changes in river geomorphology, flooding, or debris compared to the previous photo.

## Results

### Volume of Engagement

Between May 2022 and June 2024, 4474 photographs were submitted, of which 3402 (76.8%) met analytical criteria, i.e. the date the photograph was taken could be extracted from the file and the photograph was taken at the correct post.

### Public Engagement with Individual Photo Posts

**Fig. 6:**
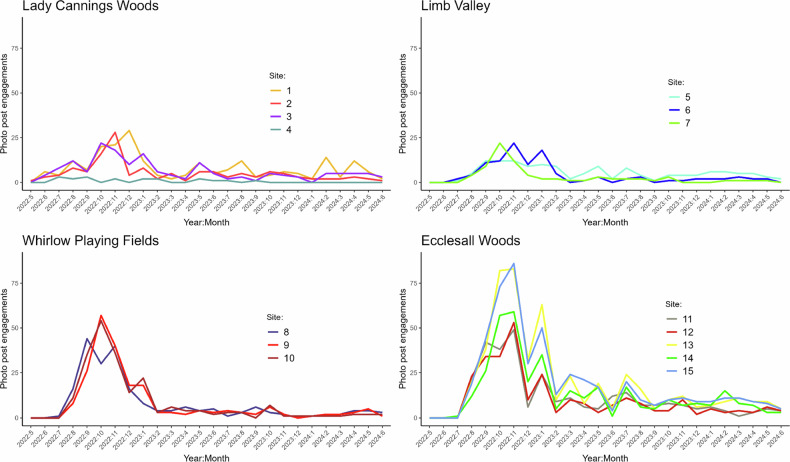
Photo post engagement over time for each region of the rivers course; **top left**) Lady Cannings Plantation, **top right**) Limb Valley, **bottom left**) Whirlow Playing Fields, **bottom right**) Ecclesall Woods (*n* = 3420)

The 15 photo posts were spread over four distinct areas: Lady Cannings plantation, Limb Valley, Whirlow playing fields and Ecclesall Woods. These areas are geographically distant and the public interacting with these spaces have different demographics. Each area’s photo posts followed a similar trend in the number of photos, with the exception of photo post 4, which is not situated on a footpath and as such had very low engagement (Fig. 6). Engagement in the citizen science project peaked for all posts between August 2022 and February 2023, with posts in Whirlow playing fields and Ecclesall Woods being the most popular.

### How the Public Engage with the Limb Brook NBS Photo Posts

To determine how individual members of the public engaged with the citizen science project, the SRWT inbox was analysed using discrete email addresses as a proxy for individual members of the public. This analysis was undertaken using AppsScript (Lacey and Andreasen 2025); no email addresses were extracted from the inbox and stored elsewhere. 648 individual email addresses were analysed, and these had engaged a total of 1771 times. Each engagement resulted in at least one photo for analysis, however, the submission of multiple photos per engagement did occur. Three types of citizen scientists were characterized (Table 1) from these results.

**Table 1:** Characterisation of citizen scientist types and their contribution (*n* = 1771)

Type of citizen scientist	Number of submitted photos	Percentage of people (number)	Percentage of email received (number)
Short and sweet	1–3	84.4% (547)	45.7% (809)
Dabbler	4–9	12.6% (82)	27.9% (494)
Hardcore	10 and over	2.3% (15)	26.4% (468)

Most citizen scientists (84.4%) were described as “Short and sweet”, contributing between once and three times within the project’s time frame. A smaller group of “Dabblers” made up 12.6% of citizen scientists who contributed a quarter of the emails received. Strikingly, a small group of 15 (2.3%) “hardcore” citizen scientists contributed an impressive 26.4% of all emails received.

#### Community Engagement and Rainfall

**Fig. 7:**
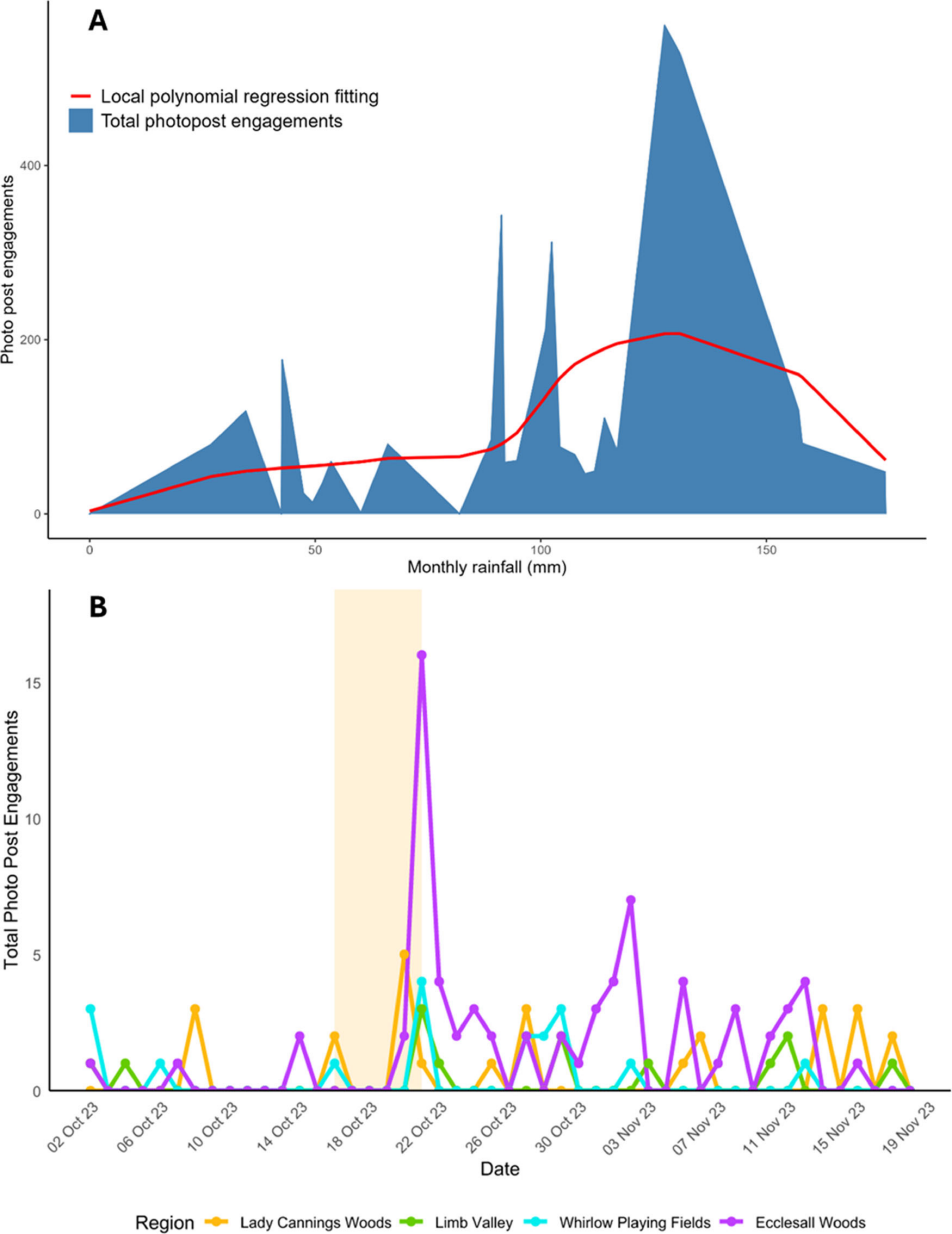
Photo post engagement and rainfall. **A** Correlation between rainfall and number of photographs across all 15 photo posts, smoothed with local polynomial regression fitting. Rainfall is measured in mm/month (*n* = 3420); **B** Impact of Storm Babet (highlighted) on photo post engagement

The Limb Brook NBS project was designed to slow the flow of water running through the Limb Brook catchment and into the River Sheaf, specifically in times of moderate and high rainfall. The photo post text stated, “*We’d love you to take pictures at this location throughout all seasons and weather conditions (especially during and post rain)*”. To determine the correlation between the public’s engagement with the citizen science photo posts and rainfall, a regression analysis was undertaken (Fig. 7A). The number of photos typically increased with rainfall up until very high rainfall events (greater than approx. 125 mm/month), when the number of photos then decreased. Analysis of photo post engagement to daily rainfall showed no correlation (data not shown).

Within the time frame of the study, the UK experienced Storm Babet; 117.4 mm of rainfall was recorded in Sheffield in four days, peaking on the 20th of October 2023, with a 139-year record of 61.4 mm rainfall being recorded (Sheffield October average monthly rainfall = 66 mm) (Sheffield City Council 2024). Engagement in photo posts was investigated before, during and after this 1 in 100-year storm event (Fig. 7B). During the highest periods of rainfall, 19th Oct onwards, an Amber Rain Warning was in place, corresponding to the limited engagement during the storm (Fig. 7B: highlighted area). Engagement with photo posts subsequently increased by ~3-fold immediately after the storm, with engagement tailed off to pre-storm levels over the next month (Fig. 6).

#### The Value of Photo Posts in Measuring the Impact of NBS

The photo posts were installed as a method to determine the impact of the NBS interventions in the Limb catchment using citizen science. The photo post text states, “*This [submitting a photo] will help us monitor how water bodies and their habitats change over time, and help us study how effective our features are in terms of slowing and storing water and supporting wildlife*”.

To this end, the photographs over the time course of the report were analysed by eye and the following impacts were determined: slowing the flow via attenuation ponds and biodiversity impact (Case Study 1), slowing the flow via leaky dams and dam failure (Case Study 2) and geomorphological impact of leaky dam installation (Case Study 3).

Professional maintenance monitoring of the leaky dams was undertaken by SRWT in the winter of 2024; the leaky dams had been in situ for ~3 years. Photographs were taken of leaky dam structures with both upstream and downstream views, as well as field notes including any accumulated woody debris and/or sediment, geomorphological changes in the river at the site of the leaky dam and any recommendations for maintenance and/or improvement of the leaky dams. Where leaky dams had been washed away was also recorded, along with possible reasons for the intervention failure. Interestingly, the professional monitoring undertaken by SRWT noted that recording longitudinal impacts by capturing before and after photos of leaky dams was difficult without employing fixed point photography mechanisms.

It is tempting to speculate that the inclusion of photo posts across more of the NBS interventions would not only increase the opportunities for public engagement and an increase in the coverage of citizen data but would also support professional monitoring, thus stacking monitoring benefits.

**Case study 1**: Slowing the flow via attenuation ponds and biodiversity impact.

**Fig. 8:**
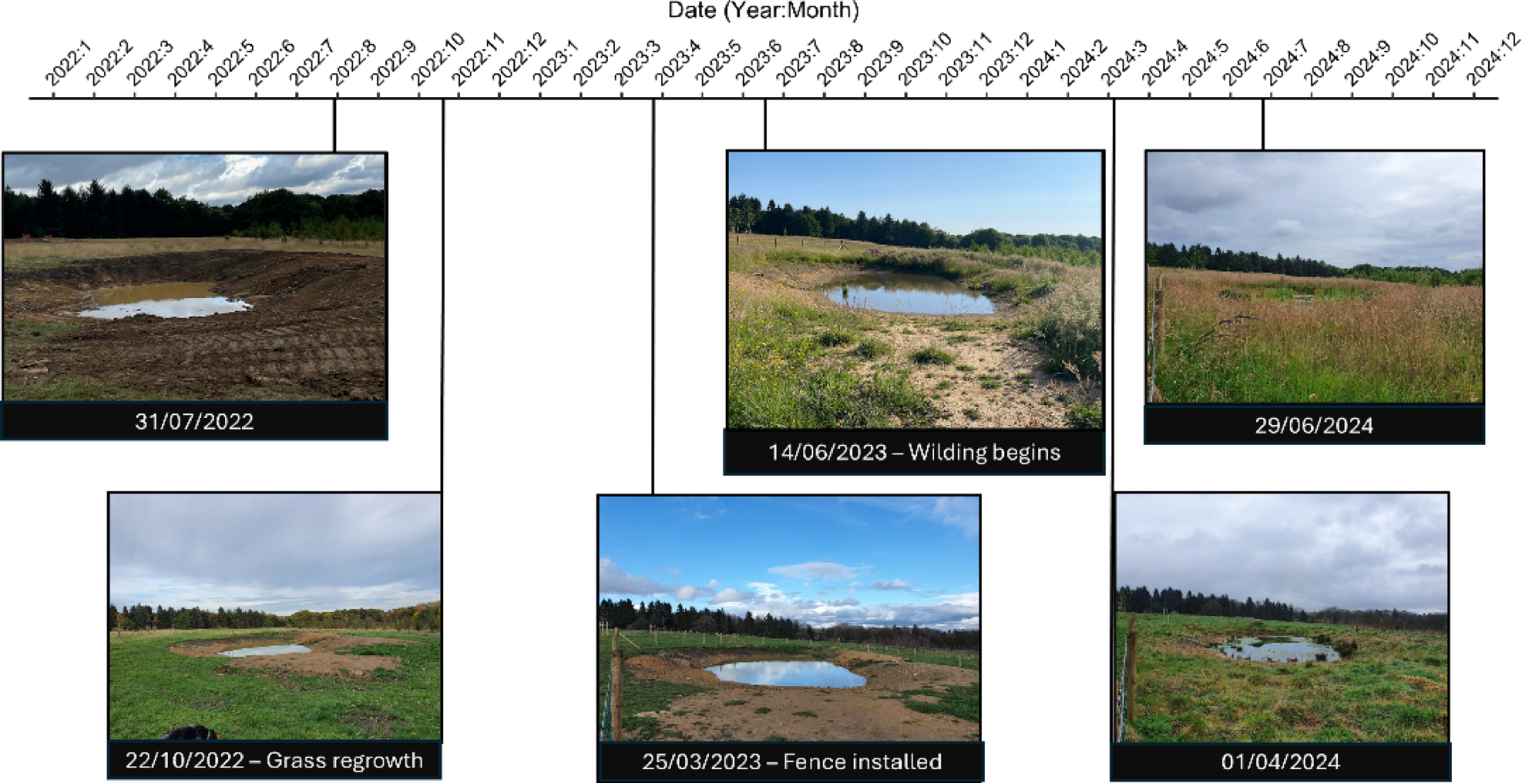
Timeline of changes to the biodiversity observed over the course of collected data for site 8. Presented images show key dates for changes in biodiversity

Site 8 was an attenuation pond that exhibited changes in biodiversity (Fig. 8). These changes differed significantly compared to other sites in the Whirlow Playing fields region. The site was fenced off around six months after the date of construction, which led to the quick establishment of wild grasses on the banks of the pond. A persistent algal bloom was also visible at the site, becoming fully established along with the grasses in late 2023. By June of 2024 the site had changed dramatically.

**Case study 2:** Slowing the flow, leaky dams and dam failure.

**Fig. 9:**
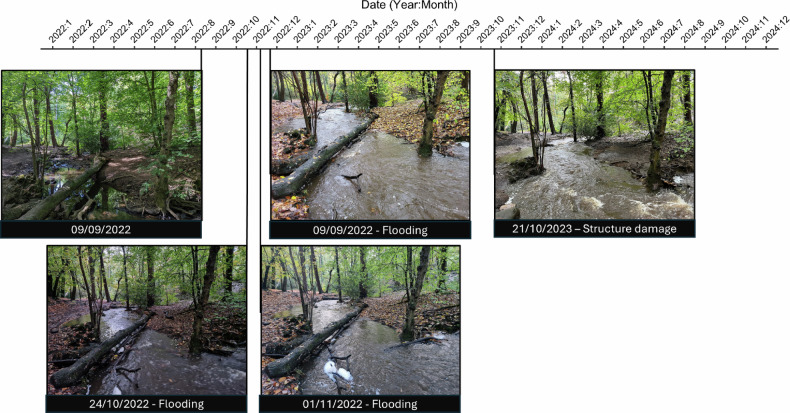
Timeline of damage to the structure installed at site 11 over the course of data collected. Presented images show high-risk events likely to weaken or damage the structure

A leaky dam structure was installed at site 11 to aid in the flow of the river during light flooding events by reducing further downstream flooding. Figure 9 shows that during flooding events on 24/10/2022, 01/11/2022, and 09/09/2022, the leaky dam structure slowed the speed and reduced the flow of water further downstream. During a larger flooding event on 21/10/2023 (Storm Babet) the structure became overwhelmed and was dislodged, washing further downstream.

**Case study 3:** Geomorphological impact.

**Fig. 10:**
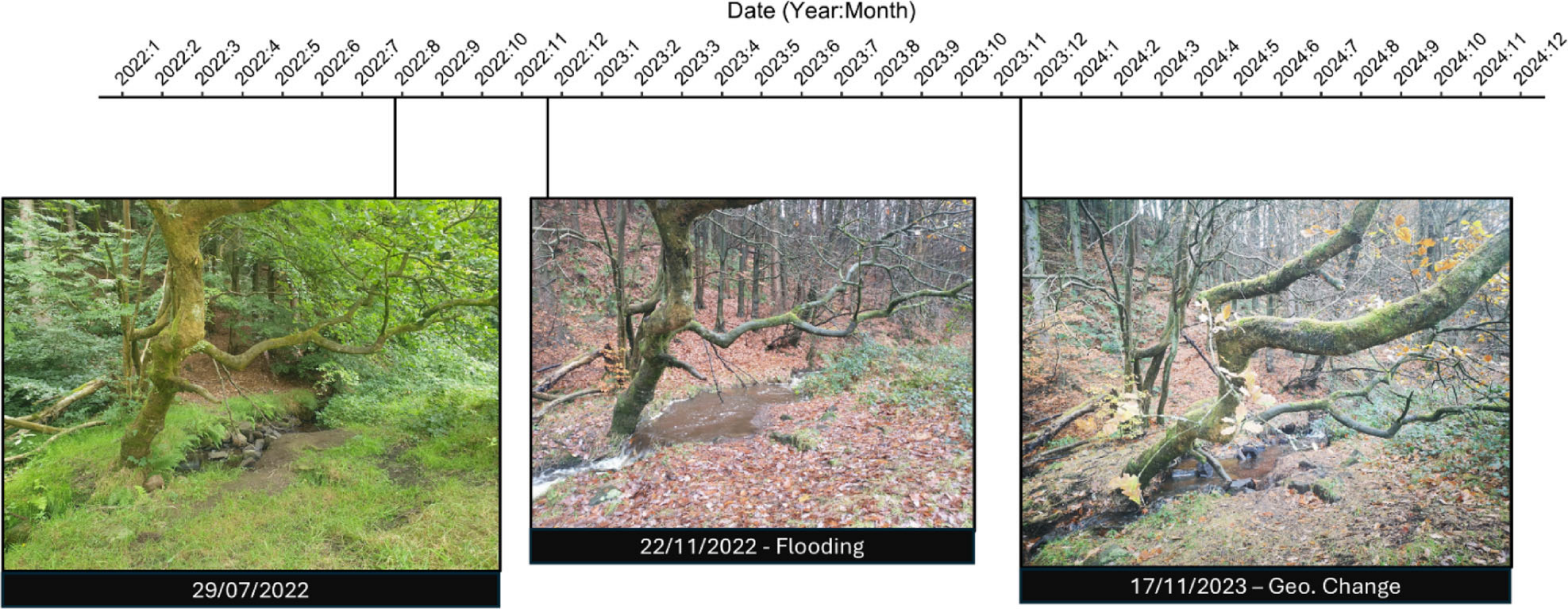
Timeline of changes caused by erosion to the riverbed and banks at site 6 over the course of data collection. Presented images highlight the high-risk events and geomorphological changes

Site 6 sat at a narrower part of the stream, which was more susceptible to flooding and high-water levels than other areas. As a result, changes in geomorphology were easily noticeable over the span of images. Figure 10 shows the impact flooding events had on this site. Repeated exposure to abnormal water flow resulted in the removal of sediment and widening of the streams channel. This sediment removal exposed and weakened the roots of the tree present at the site, resulting in its partial collapse.

### Time Efficiency of NBS Intervention Maintenance Monitoring

The time efficiency of the analysis of citizen science photographs and physical monitoring by SRWT was recorded and compared.

To analyse the citizen science photographs, emails were named, organised and in photo post-specific folders by Google Apps Scripts (section “Data processing and analysis”). These scripts were designed to take less than 6 min to undertake each function on 4474 photographs. The results folders corresponding to each photo post contained photographs in chronological order. As each photograph was reportedly taken from a fixed point, the photographs with poor quality images or of the incorrect location could be easily observed and deleted, with ~4474 photographs verified in two and a half hours. Simple analysis, such as if a leaky dam had been washed away, took a few minutes, with the timescale of the event easily identified by the time encoded with the file name. Taken together, determining the approximate date of intervention failures over the 26 months of the project took 3 h. It is important to acknowledge the unseen labour of citizen scientists, as the time taken for members of the public to submit photographs is not known, especially for those “Hardcore” citizen scientists who engage frequently (Table 1).

In contrast, in-person monitoring by SRWT across all NBS intervention and photo post sites took a full day for simple observations, such as whether a leaky dam had been washed away; maintenance monitoring, including photographs and field notes, took two days with two members of staff.

Comparison between the two methods of NBS maintenance monitoring is not straightforward, as they provide different data within different time scales with different time efficiencies. That said, the time efficiency of the analysis of citizen science-generated photographs to answer simple questions is a clear strength of this methodology.

## Discussion

The Limb Brook NBS project demonstrates both the promise and the challenges of using citizen science photo posts to monitor natural flood management interventions. Analysis of more than 4500 images submitted over 22 months shows how participatory monitoring can generate valuable insights into hydrological processes, ecological succession, and the structural performance of interventions such as leaky dams. Unlike most previous citizen science studies, which have focused primarily on biodiversity monitoring, this case highlights hydrological and structural processes, areas that remain underrepresented in the citizen science literature. At the same time, the project provides lessons for the future design of citizen science initiatives in environmental governance (Nardi et al. 2021).

One of the clear strengths of the approach was the scale of community participation. The volume and temporal distribution of submissions indicate that the public not only engaged with the scheme but also acted on its objectives, with higher contributions during rainfall events (Fig. 7). This aligns with evidence from wider citizen science literature that situational relevance and perceived scientific value can sustain engagement (Sauermann et al. 2015). Importantly, the typology of contributors, ranging from casual “short and sweet” participants to highly committed “hardcore” volunteers, reflects the diversity of motivations that citizen science projects must accommodate. Moreover, the imagery provided actionable insights, enabling project managers to identify issues such as dam failures and sediment accumulation and to document seasonal changes in pond water levels. This supports arguments from environmental governance literature that citizens can play an active role in adaptive management, contributing not only data but also local knowledge and observational continuity (Landstrom et al. 2011, Landstrom et al. 2019).

However, several limitations emerged that constrained the long-term effectiveness of the approach. Engagement declined steadily after the initial installation of posts, reflecting patterns of volunteer fatigue observed in other citizen science projects (Conrad and Hilchey 2011, Walker et al. 2021). The absence of systematic feedback mechanisms meant that contributors could not easily see the impact of their efforts, reducing incentives for continued participation (Walker et al. 2021). The reliance on email submissions also created administrative burdens and limited opportunities to communicate with participants under data protection regulations. From a technical perspective, some posts were poorly positioned, limiting their ability to capture relevant hydrological features or providing insufficient reference markers (e.g., depth gauges) to quantify change. These limitations highlight the need to pair citizen science imagery with conventional monitoring methods to ensure data robustness and interpretive depth. They also raise broader ethical questions about sustaining unpaid public labour within monitoring frameworks (Woodcock et al. 2017).

Despite these challenges, the Limb Brook case offers opportunities for innovation. The popularity of the photo posts indicates that they can serve as a gateway to broader citizen engagement in environmental monitoring. Building on this enthusiasm could involve developing digital platforms that allow real-time uploads, automated tagging, and visible feedback loops. Similar platforms have proven successful in biodiversity monitoring (e.g., Callaghan et al. 2022, Schubert et al. 2019), and their adaptation to hydrological citizen science could transform engagement dynamics. However, digitising risks excluding those without access to smartphones or digital literacy, possibly creating digital inequalities (Jönsson et al. 2024).

From a practical standpoint, photo posts provide a cost-effective means of extending the observational capacity of project managers, particularly for identifying failures or blockages between scheduled site visits. A clear example of this is within the monitoring of leaky dams. Here, Storm Babet (October 2023) was seen to wash away the leaky dam in Case study 2 (Fig. 9) via the increased engagement with photo posts (Fig. 7), well before the professional maintenance monitoring by SRWT in the winter of 2024. This “eyes on the river” model enhances responsiveness and resilience, especially when linked to active community groups capable of rapid on-the-ground intervention. Advances in artificial intelligence (AI) and machine learning further expand the potential of this approach, offering tools for automated image classification and temporal analysis of citizen-generated data. Nevertheless, such applications require critical evaluation to ensure data quality, transparency, and accountability in the use of volunteer contributions (Wiggins and Wilbanks 2019).

At the institutional level, sustaining citizen science engagement presents structural challenges. Effective coordination and communication demand resources that often exceed the short-term funding cycles typical of NBS projects. Without continuity, projects risk alienating volunteers and undermining trust in future initiatives (Frantzeskaki et al. 2019). Moreover, the inherent limitations of photographic monitoring such as restricted resolution, perspective, and calibration mean that citizen-generated data cannot replace professional hydrological surveys. Rather, they should complement formal methods, adding temporal and spatial coverage while fostering social legitimacy. Finally, institutional and funding shifts may weaken long-term monitoring commitments, leading to data underuse or loss. These challenges highlight the vulnerability of citizen science to policy discontinuities and the contested politics of environmental governance (Gobel 2019). Embedding citizen participation into enduring monitoring frameworks, supported by dedicated coordination and evaluation capacity, will be essential to address these risks.

The decline in engagement after the initial six months reflects a documented pattern of participation fatigue in citizen science (Geoghegan et al. 2016). However, this should be understood less as an inherent limitation and more as a design and infrastructural challenge. Sustained engagement depends on reinforcing participant value, visibility, and relevance.

Several strategies emerge. First, the absence of feedback loops was a critical barrier; future approaches should incorporate visible and timely feedback (e.g. dashboards or public-facing platforms) so participants can see how their contributions are used. Second, reliance on email submissions limited interaction and created inefficiencies; integrated digital platforms could enable real-time uploads and two-way communication. Third, engagement should be reframed as a tiered participation ecosystem, reflecting different levels of commitment from occasional contributions to more sustained involvement. Fourth, embedding citizen science within existing social infrastructures (e.g. schools or community groups) can provide continuity beyond project lifecycles. Finally, long-term funding and governance are essential, as short-term project cycles are misaligned with the temporal demands of both NBS monitoring and community engagement. Overall, sustaining participation of citizen scientists is less about public willingness and more about designing durable socio-technical systems that integrate participation, feedback, and governance.

In summary, the Limb Brook photo post project illustrates both the promise and the constraints of citizen science in the monitoring of NBS interventions. The findings reinforce the view that citizen science should not be regarded merely as a low-cost alternative to professional monitoring, but as a complementary mechanism that can enhance data richness, strengthen community ownership, and provide early-warning insights. This study contributes new empirical evidence on the use of citizen-generated imagery for hydrological monitoring, while also extending conceptual debates on governance by showing how citizen science can both support and challenge institutional monitoring frameworks. To realise these benefits, careful attention must be paid to project design, digital infrastructure, participant feedback, and long-term funding strategies.

## Conclusion

Citizen science offers a valuable and scalable supplement to formal hydrological monitoring within nature-based flood management. The findings of this study complement emerging monitoring approaches such as low-cost sensing and UAV-based methods, highlighting the value of citizen science as part of hybrid monitoring systems. The Limb Brook project demonstrates that even simple visual data, when collected consistently, can inform infrastructure assessment, biodiversity tracking, and adaptive management. Integrating such participatory approaches into existing institutional frameworks can enhance the resilience, transparency, and inclusivity of environmental governance.

To sustain these benefits, structural barriers such as fragmented data systems, limited participant feedback, and short-term funding must be addressed. Embedding citizen science within long-term NBS monitoring frameworks, supported by digital innovation and sustained coordination, could significantly advance the co-production of knowledge and strengthen local ownership of climate adaptation strategies. Future work should move beyond short-term participatory experiments towards the design of persistent citizen sensing infrastructures, where communities are not only contributors of data but active participants in long-term environmental governance.

## Data Availability

The photographs that support the findings of this study are available from Sheffield and Rotherham Wildlife Trust, but restrictions apply to the availability of these data due to GDPR and thus are not publicly available. The data are, however, available upon request and with the permission of Sheffield and Rotherham Wildlife Trust.
